# Post genome-wide association studies functional characterization of prostate cancer risk loci

**DOI:** 10.1186/1471-2164-14-S8-S9

**Published:** 2013-12-09

**Authors:** Junfeng Jiang, Weirong Cui, Wanwipa Vongsangnak, Guang Hu, Bairong Shen

**Affiliations:** 1 Center for Systems Biology, Soochow University, Jiangsu 215006, China

**Keywords:** Genome-wide association study, prostate cancer, gene ontology, pathway, network

## Abstract

**Background:**

Over the last decade, genome-wide association studies (GWAS) have discovered many risk associated single nucleotide polymorphisms (SNPs) of prostate cancer (PCa). However, the majority of the associated PCa SNPs, including those in linkage disequilibrium (LD) blocks, are generally not located in protein coding regions. The systematical investigation of the functional roles of these SNPs, especially the non-coding SNPs, becomes very necessary and helpful to the understanding of the molecular mechanism of PCa.

**Results:**

In this work, we proposed a comprehensive framework at network level to integrate the SNP annotation, target gene assignment, gene ontology (GO) classification, pathway enrichment analysis and regulatory network reconstruction to illustrate the molecular functions of PCa associated SNPs. By LD expansion, we first identified 1828 LD SNPs using 49 reported GWAS SNPs as a start. We carefully annotated these 1828 LD SNPs via either UCSC known genes, UCSC regulation elements, or expression Quantitative Trait Loci (eQTL) data. As a result, we found 1154 SNPs were functionally annotated and obtained 205 unique PCa genes for further enrichment analysis. The enriched GO biological processes and pathways were found mainly related to regulation of cell death, apoptosis, cell proliferation, and metabolic process, which have been proved essential to cancer development. We constructed PCa genes specific transcription regulatory networks, finding several important genetic regulators for PCa, such as *IGF-1/IGF-2 *receptors, *SP1, CREB1*, and androgen receptor (*AR*).

**Conclusions:**

A comprehensive framework was proposed for integrative and systematic analysis of PCa SNPs, the analysis can provide essential information for the understanding of the regulatory function of GWAS SNPs in PCa, and will facilitate the discovery of novel candidate biomarkers for diagnosis and prognosis of PCa.

## Background

As one of the most common but complex malignancy in men of developed countries, prostate cancer (PCa) has been the second death-leading one among various cancers [1-4]. However, the pathophysiology and molecular mechanism for PCa have remained poorly understood. According to the National Human Genome Research Institute (NHGRI) Catalog of published genome-wide association studies (GWAS) [5], there have been 49 SNPs reported in 14 publications (Caucasian population, as of June 3, 2011) relevant to PCa. Although these comprehensive studies have elucidated the mechanism of incidence of PCa to a certain extent, limited conclusions have been made regarding the causal correlation between the identified SNPs and the molecular carcinogenesis of PCa [6]. Moreover, findings from GWAS cannot directly lead to the identification of disease associated genes. One way is to consider genes overlapped by the originally identified SNPs as functional markers to complex disease traits [7]. Through this approach, several genes have been reported to be associated with PCa, such as *TCF2 *[8], *HNF1B *[9], *MSMB *[9], and *EHBP1 *[10]. However, most of the PCa GWAS SNPs were found to be located in intergenic region [5,11-16], making it hard to characterize the biological function at the gene level.

Gene expression has been reported to play essential roles in numerous important biological process and is highly heritable [17]. Considering the SNPs may have functional impacts on gene expression, the expression Quantitative Trait Loci (eQTL) approach has been proposed and commonly used to facilitate the identification of associations between intergenic SNPs and traits [18-20]. To date, several studies have demonstrated the great power of the eQTL approach to detect SNPs with stronger effects on gene expression from various human samples, including lymphoblastoid cell lines (LCLs) [19,21-28], monocytes [29], lymphocytes [30], adipose [31], brain [32], and liver [33]. However, those eQTL SNPs are also reported to act in a tissue-specific manner [34,35]. In this study, we mainly chose eQTLs of LCLs or those reported similar to LCLs [36-38], such as monocytes [29], and lymphocytes [30], which may provide much more information than LCLs alone.

Despite the significant power of genetic mapping in complex traits using eQTLs approach, there are many other factors may affect gene expression, such as the transcript stability, epigenetic effects, environmental stimuli, drug exposure, populations, and clinical covariates [17,36,39,40]. So far, most of PCa marker studies mainly focus on single genes, while how the discovered genes interact together to exert a significant combined effect on PCa still remains elusive. Recent studies [41,42] indicate that genes with altered expression levels may individually contribute a moderate risk to disease, but act in a synergistic mode at biological pathway or gene-network level [43,44]. Methods that focus on pathway/network rather than individual genes can detect significant coordinated changes. A representative analytic approach is the Gene Set Enrichment Analysis (GSEA), which has been commonly adapted [3,4,45-49] to sort the collected genes into predefined pathways or functional categories.

In this study, first, we performed a comprehensive assessment of the potential function of PCa related SNPs, utilizing the Encyclopedia of DNA Elements (ENCODE) genomic annotation databases, the annotation systems from University of California Santa Cruz database (UCSC table browser; http://genome.ucsc.edu/), and knowledge of PCa specific transcription factor binding sites (TFBS), e.g., *AR, ER*, and *FoxA1*, defined by previous studies [50,51]. Then, we collected the Pca related genes by either overlaying the SNPs or eQTL mapping. Functional enrichment analysis of the collected genes was then performed using Gene Ontology (GO) and predefined canonical pathways encoded in MetaCore™ (GeneGo, Inc.), a commercial integrated knowledge database. Finally, PCa-specific transcription regulatory networks were constructed from the inferred gene set. Our work may provide a practical framework for integrative genomics analysis of PCa at system level, which may provide a better insight into PCa and other complex diseases.

## Results

### Identification and annotation of SNPs in LD with GWAS PCa risk SNPs

**Figure 1:**
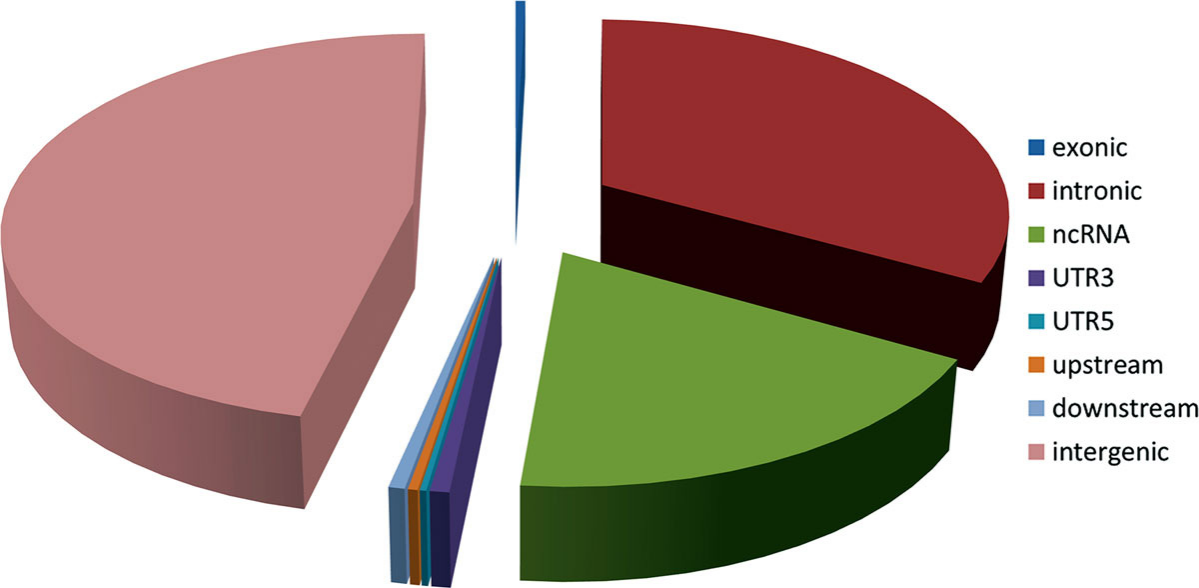
**Pie-chart of genomic distributions of the 1828 PCa SNPs**. Labels with different colors were used for clarity.

We identified a total of 1828 SNPs in LD with the 49 reported GWAS PCa SNPs (Additional file 1). All SNPs were mapped to SNPs database (NCBI36/hg18 assembly, Mar. 2006, UCSC) to extract the information of alleles. Then the UCSC Known Gene annotation encoded in ANNOVAR [52] was processed on the identified 1828 SNPs. The results of the annotation were provided in Additional file 2, in which the first column explained the genomic locations of the SNP, e.g., exonic, intronic, splicing, ncRNA, 3'UTR, 5'UTR, upstream, downstream or intergenic, with the corresponding gene or the flanking gene symbols in the second column. Of the 1828 SNPs, 8, 599, 377, 4, 12, 6, and 10 SNPs were found in exon, intron, ncRNA, 5'UTR, 3'UTR, upstream, and downstream, respectively, while the rest 852 SNPs were located in intergenic regions, which was demonstrated as a pie-chart in Figure 1. On the other hand, we also collected regulatory data from three UCSC tracks, Yale TFBS, broad histone, and regulatory elements, as well as binding sites of *AR/ER/FoxA1 *defined by previous studies [50,51] (Additional file 3), to analyze all 1828 SNPs. As a result, 284 SNPs were annotated (Additional file 4), including 86 intergenic SNPs. Since the regulatory data were still under power to interpret the intergenic SNPs and could not directly lead to the corresponding gene, we mapped the all 852 intergenic SNPs to eQTL data (see Materials and Method part), and identified 151 unique genes corresponding to 138 SNPs.

### Enrichment analysis for PCa candidate genes

In total, we compiled a set of 205 unique PCa candidate genes, including 41 genes from ANNOVAR annotation using UCSC known genes, 151 genes by eQTL mapping, and 33 genes reported by the 14 GWAS publications, for enrichment analysis. To explore the synergistic function among the PCa genes, GO enrichment was performed using GeneGO. The top 10 terms of the three GO functional ontologies were listed in Table 1. The most significant term in each category was: regulation of cell death in biological process (*p*-value = 4.944 × 10^-9^), insulin-like growth factor receptor binding in molecular functions (*p*-value = 6.617 × 10^-18^), and cytoplasmic part in localizations (*p*-value = 1.009 × 10^-6^). Other significant terms of interest were also high-lighted, including regulation of apoptosis, metabolic process, insulin receptor binding, hormone activity, and NADH dehydrogenase complex. We also performed a pathway enrichment test for these 205 PCa genes, utilizing pre-defined canonical pathway sets from GeneGO system. As shown in Table 2, 8 pathways belong to oxidative, ubiquinone, transcription, development, cell adhesion, and cell cycle were significantly enriched with our PCa genes, including phosphorylation (*p*-value = 3.187 × 10^-4^), metabolism (*p*-value = 4.790 × 10^-4^), CREB pathway (*p*-value = 7.620 × 10^-4^), etc.

**Table 1:** The top 10 terms of GO functional ontologies.

GO terms	Observed*	*p*-value^#^
*GO Biological Processes*		
Regulation of cell death	40	4.944 × 10^-9^
Regulation of apoptosis	39	5.506 × 10^-9^
Regulation of programmed cell death	39	6.945 × 10^-9^
Response to external stimulus	39	1.237 × 10^-8^
Metabolic process	133	2.357 × 10^-8^
Epithelial cell proliferation	9	7.699 × 10^-8^
Response to organic cyclic compound	19	1.153 × 10^-7^
Negative regulation of biological process	61	1.650 × 10^-7^
Rhythmic process	15	2.056 × 10^-7^
Branching morphogenesis of a tube	12	2.070 × 10^-7^

*GO Molecular Functions*		
Insulin-like growth factor receptor binding	11	6.617 × 10^-18^
Insulin receptor binding	10	1.501 × 10^-12^
Protease binding	11	1.685 × 10^-11^
Binding	156	1.574 × 10^-10^
Protein binding	115	1.593 × 10^-9^
Protein complex binding	17	2.310 × 10^-7^
Hormone activity	10	4.671 × 10^-6^
Catalytic activity	76	3.102 × 10^-5^
Oxidoreductase activity, acting on NADH or NADPH, quinone or similar compound as acceptor	5	6.596 × 10^-5^
4 iron, 4 sulfur cluster binding	4	8.215 × 10^-5^

*GO Localizations*		
Cytoplasmic part	99	1.009 × 10^-6^
Mitochondrial envelope	19	2.992 × 10^-6^
Mitochondrial part	22	9.733 × 10^-6^
Envelope	23	1.480 × 10^-5^
Mitochondrion	34	2.505 × 10^-5^
Organelle envelope	22	3.432 × 10^-5^
Mitochondrial inner membrane	13	7.758 × 10^-5^
Mitochondrial respiratory chain complex I	5	7.937 × 10^-5^
NADH dehydrogenase complex	5	7.937 × 10^-5^
Respiratory chain complex I	5	7.937 × 10^-5^

* Number of the observed genes of the gene list in the category

**^# ^***p*-values were calculated by FDR (0.05) adjustment

**Table 2:** The enriched GeneGO canonical pathways.

Category	GeneGO canonical pathways	*p*-value^#^
Oxidative	Phosphorylation	3.187 × 10^-4^
Ubiquinone	Metabolism	4.790 × 10^-4^
Transcription	CREB pathway	7.620 × 10^-4^
Development	WNT signaling pathway. Part 1. Degradation of beta-catenin in the absence WNT signaling	5.859 × 10^-4^
Cell adhesion	ECM remodeling	1.119 × 10^-3^
Cell cycle	Role of SCF complex in cell cycle regulation	2.073 × 10^-3^
Cell cycle	ESR1 regulation of G1/S transition	3.018 × 10^-3^
Cell cycle	Regulation of G1/S transition (part 1)	4.521 × 10^-3^

^# ^*p*-values were calculated by FDR (0.05) adjustment

### PCa-specific transcription regulatory networks

**Figure 2:**
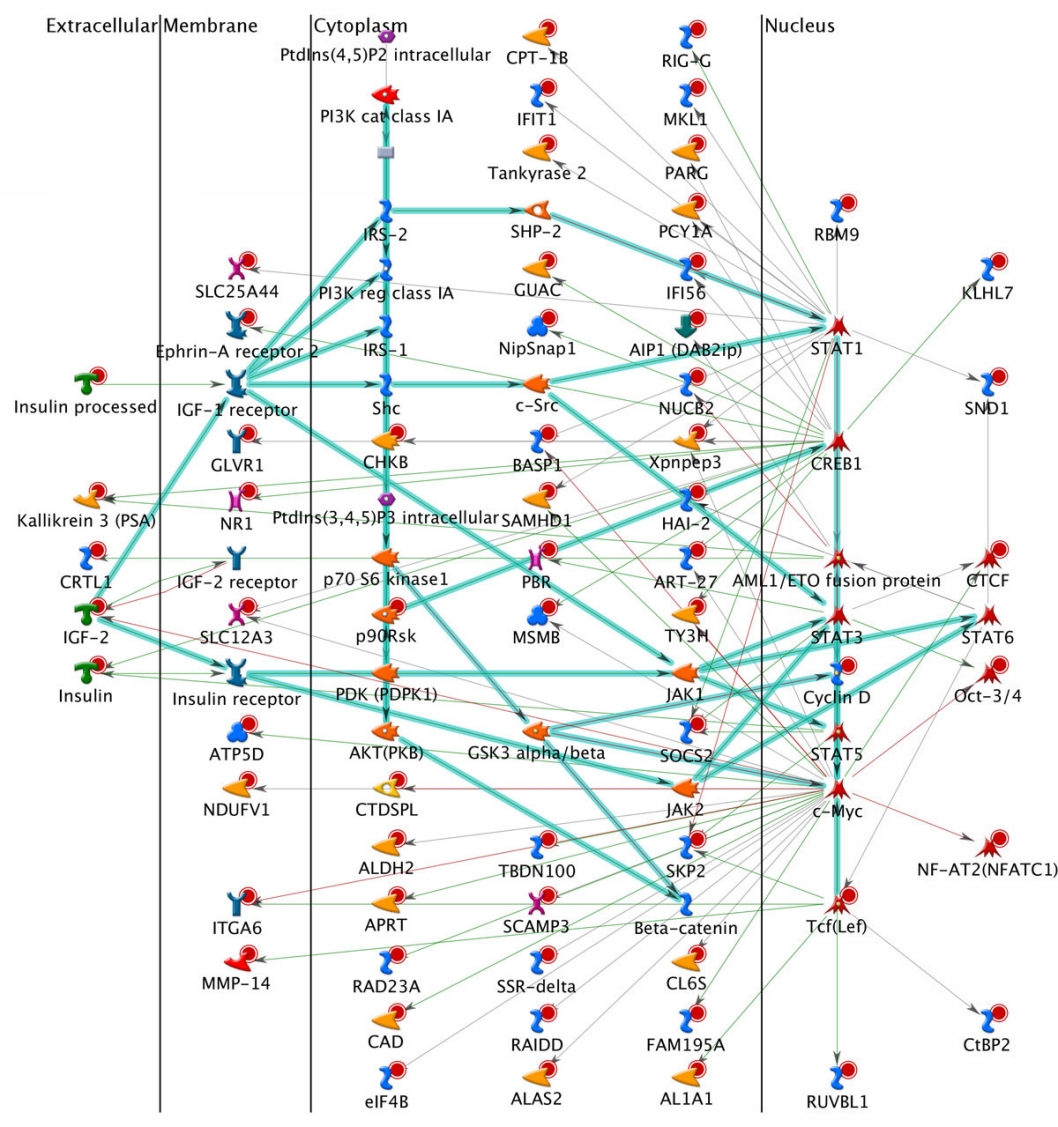
**Network map of complex pattern of AML1/ETO fusion protein, IGF-1 receptor, Insulin receptor and IGF-2 receptor**. Legends of nodes (genes) and edges (interaction) of the network were illustrated in Additional file 6.

Using the network construction module in GeneGO, we constructed the putative significant PCa-specific networks with determined *p*-values. Of the 205 PCa genes, 18 genes were failed to be enriched with any transcription regulatory network. Specifically, by retrieving the additional nodes such as interconnected genes or transcription factors, the top 10 significant transcription regulation networks were shown in Additional file 5. In the constructed transcription regulation networks, the most significant one was a network connecting *AML1/ETO *fusion protein, *IGF-1 *receptor, Insulin (*INS*) receptor, and *IGF-2 *receptor. 64 of 99 genes in this network are in our PCa candidate gene list (named as seed nodes) and the other 35 genes were recruited from the interaction database from GeneGO. It was obvious that most of the genes in this network were highly enriched in cellular metabolic process (45.3%, *p*-value = 6.947 × 10^-21^) and peptide stimulus (25.3%, *p*-value = 2.680 × 10^-21^), which had been proved significantly associated with the pathophysiology hypothesis of PCa incidence [53-55]. We visualized the network map in Figure 2, in which the genes were classified into four localizations: extracellular, membrane, cytoplasm, and nucleus, to clearly illustrate the activities of cellular process. Of note, different symbols were used to represent different genes, which were listed in Additional file 6 (Nodes sheet). And each color was assigned to the different mechanism between two genes, for example, green lines implied activation, brown implied inhibition, and grey indicated unspecified mechanism (Additional file 6, Interactions sheet). To better describe the importance of collected PCa genes, we highlighted the edges connecting critical nodes in this network, such as *IGF-2, IGF-1, IRS-1, INS, STAT1, CREB1, STAT3, STAT5, c-Myc *and *Tcf*. Moreover, we used GeneGO to conduct the statistical analysis to identify the important interactions, hubs, transcription factors, and receptors, which was listed in Additional file 6 (note, there were different sheets in Additional file 6). In this network, we identified 19 hubs, of which 11 are transcription factors, including *c-Myc, CREB1, STAT1*, etc. (Additional file 6, Hubs sheet), which is in good agreement with our annotation results of Yale TFBS. As indicated by Additional file 5, it is obvious that *IGF-1/IGF-2 *receptors were highly involved in PCa specific transcription regulation networks, and in fact, these two receptors were certainly involved in the development of PCa [56], indicating great importance of two genetic regulators. Another useful module in GeneGO is the disease enrichment approach, which can directly point out whether the constructed network is associated with studied disease, e.g., PCa in our work. After enrichment test, we found that 3 of the top 10 networks were significantly associated with PCa, that is "*HNF3, IGF-1 *receptor, *IGF-2 *receptor" (*p*-value = 1.610 × 10^-17^), "*TEF, IGF-1 *receptor, *IGF-2 *receptor (*p*-value = 3.920 × 10^-11^)" and "*COUP-TF, IGF-1 *receptor, *IGF-2 *receptor (*p*-value = 1.148 × 10^-11^)", which greatly raised the feasibility of our method.

**Figure 3:**
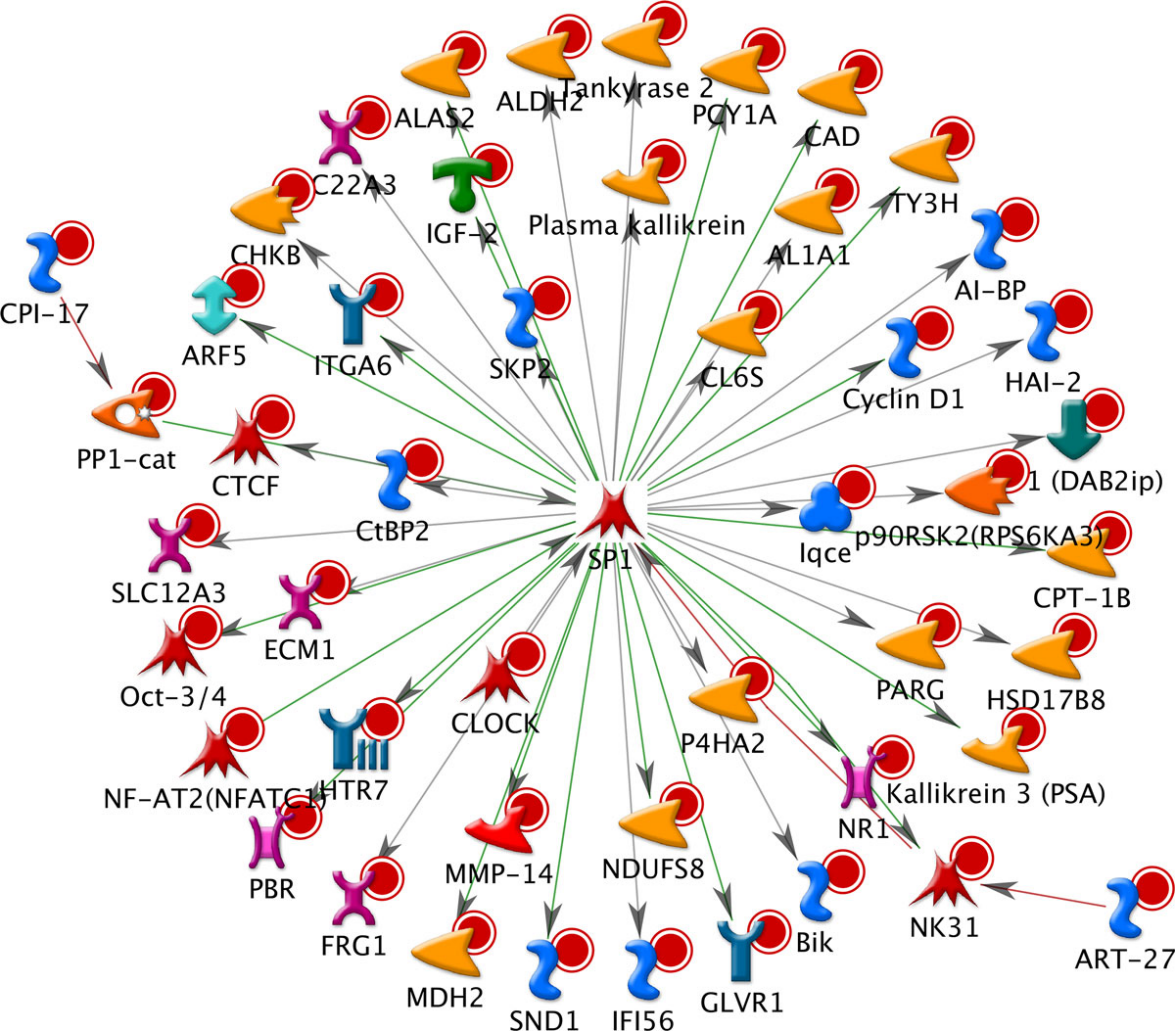
**Network map of transcription factor SP1**.

To examine which genetic factor, e.g., transcriptional factor, involve in PCa genes, we also constructed the transcription factor networks. As shown in Additional file 5, the most significant transcription factor network among the top 10 significant ones was "*SP1*", which was comprised of 48 PCa genes and 1 GeneGO gene. We visualized this network in Figure 3. Note that different symbols represent different genes, which were shown in Additional file 6, while green and grey arrow lines in the figure displayed activation and inhibition interaction between the two genes, respectively. Interestingly, most of transcription factor networks were reported to be related with PCa, such as *SP1 *[57], *c-Myc *[58], *AR *[59], and *p53 *[60], supporting the hypothesis that the collected genes from systematic annotation of PCa GWAS LD SNPs were putative PCa biomarkers, and therefore provided more informative insights for regulatory systems in PCa, rather than the analysis of SNPs alone.

## Discussion

Although PCa risk-associated SNPs identified from GWAS have been extensively investigated, the study of their synergistic function of the SNPs still remains elusive. One practical approach is to map the SNPs to related genes, which can be utilized for further systematic studies. Preliminary annotation of all 1828 LD SNPs indicated that nearly 50% of the SNPs were located in gene desert region. After collecting the 205 unique genes by gene overlapping or eQTL mapping, we further studied their biological functions by performing GO and pathway enrichment test, and constructing regulatory networks. The whole pipeline may offer us new insights into the function of PCa SNPs, genes, and relevant regulatory networks.

**Figure 4:**
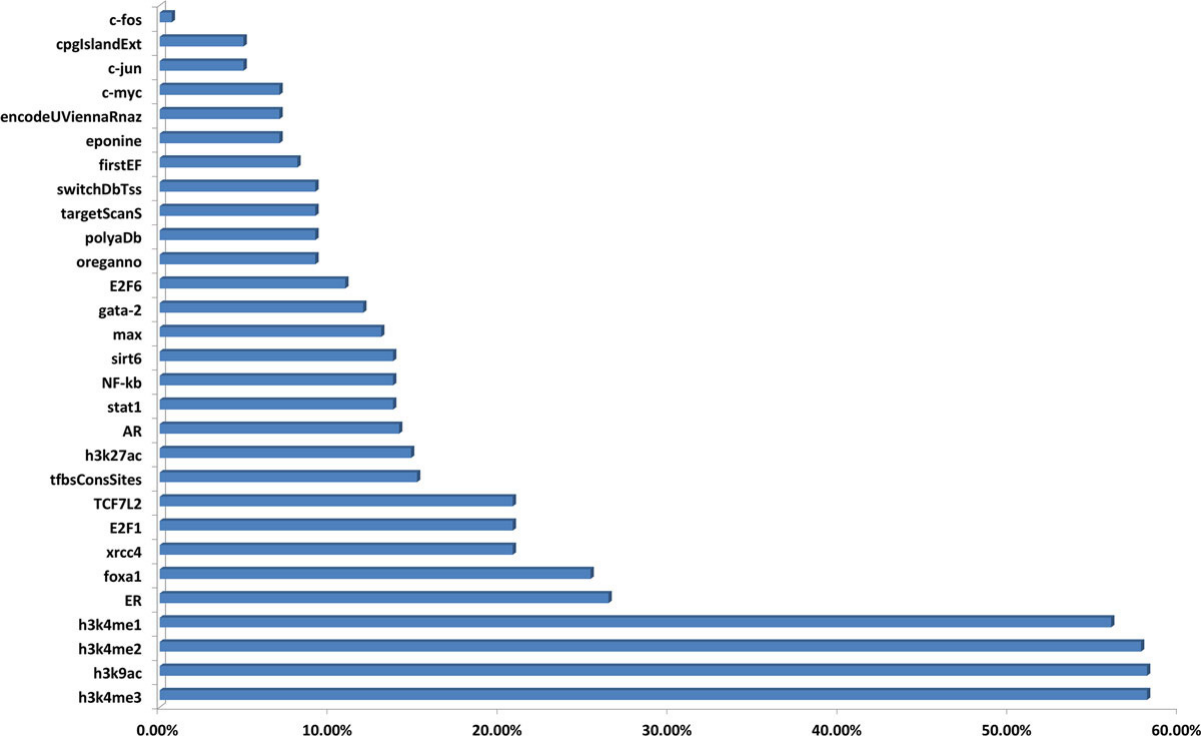
**Functional annotation of PCa SNPs using UCSC annotation tracks and *AR/ER/FoxA1 *TFBS data**. 29 regulators were analyzed and ranked.

Our SNP annotation and eQTL analysis showed that 1154 of the 1828 SNPs had been functionally interpreted with corresponding genes. We found that these 1154 SNPs were distributed in 40 SNP blocks, which was listed in Additional file 7. 8q24 has long been confirmed as a susceptible locus of PCa [9,12-14,61]. In our study, it was obvious that more SNPs (265) were located on chromosome 8 (chr8), compared to chr10 (185), chr4 (180), chr17 (157), chr2 (118), and chr3 (112), indicating that regulatory regions in chr8 might be more sensitive and critical in prostate cancer. We also found that 284 of the identified 1828 SNPs were located in genomic regions of 29 pre-defined well-known regulators from UCSC annotation tracks. As shown in Figure 4, it was obvious that most of regulators showed a moderate enrichment, while, interestingly, ~60% of 284 annotated SNPs were associated with epigenetic functions, such as methylation and acetylation of histone H3 (enhancer: h3k4me1 and h3k4me2, transcriptional activity: h3k4me3 and h3k9ac). This indicated that epigenetics might play a crucial role in the mechanism of PCa, although further experimental investigation is required.

Our GO enrichment analysis of the collected genes revealed the significant biological associations with PCa. The enriched GO biological process terms were mostly related with cellular regulation and metabolic process. In terms of biological process, we found that some genes of these GO enriched PCa genes have already been proved important in PCa, such as NADH dehydrogenase (*NDUF*) subcomplexes [52] (*NDUFA11, NDUFA13, NDUFA3, NDUFS8, NDUFV1*), *IGF-2 *[58] and *CTCF *[62] in the cellular regulation process, and *SOCS2 *[63], *HNF1B *[64], *CCND1 *[61], and *INS *[65] in the metabolic process. While top enriched terms in molecular function indicated that our PCa genes might actively involve in transcription factor binding activities. Although not surprising, the identified significant GeneGO pathways were previously reported important pathways in PCa, such as phosphorylation [66], metabolism [67], CREB signaling pathway [68-70], Wnt signaling pathway [71-73], and ECM remodeling [74].

Networks allowed us to explore the systematic gene interactions involved in cell signaling and metabolism, from initial receptor-ligand interactions to second messenger and signal transduction cascades. Interestingly, we observed a strong enrichment in transcription regulation and transcription factor networks, involving important genetic regulators, such as *IGF-1, IGF-2, SP1, c-Myc, AR*, and *p53*. In summary, our approach was efficient to discover the putative PCa associated genes using the reported GWAS SNPs as a start and public annotation data, e.g., known genes and eQTLs. Another advantage of our work is that we do not require the raw genotyping data, thus less computational burden.

Nevertheless, our works still has a few limitations. First, the number of reported GWAS SNPs is increasing according to the NHGRI website, our analysis need to be updated in the future with the changing. Second, we paid more attention to PCa genes and involved regulatory networks rather than the SNPs, which might ignore the importance of SNPs themselves, e.g., corresponding mutations at SNP sites in gene region. Third, we used LCL related eQTLs data for SNPs mapping in this study, while eQTLs derived from prostate tissue should be more comprehensive, which will be considered if data are available in the future. Fourth, the PCa specific networks were constructed based on GeneGO database. Although the quality of the databases has been validated, the interactions in the network are still with the accumulation of the scientific findings. Fifth, our work was carried out based on our computational strategies, which required further experimental validation. However, the work here explains a part of the intergenic SNPs, and therefore provides a practical and effective framework to the annotation of disease associated SNPs, especially the intergenic SNPs, at systems biology or network level.

## Conclusions

We developed a systems biology framework to evaluate the function of PCa GWAS SNPs and their synergistic biological function in PCa. We explored well defined annotation data from UCSC tracks and eQTL from publications to collect the putative PCa specific genes. Our approach offered a comprehensive analysis including GO enrichment, pathway enrichment, and network construction, providing informative insights for further study of PCa, and could be applied to other complex diseases.

## Materials and methods

### Definition and annotation of SNPs in linkage disequilibrium (LD) with the GWAS PCa risk associated SNPs

All SNPs in linkage disequilibrium (LD) (*r*^2 ^≥ 0.5) with the 49 GWAS PCa SNPs (reached genome-wide significance level with a p-value ≤ 10^-5^) were obtained from SNAP database (http://www.broadinstitute.org/mpg/snap/ldsearchpw.php; proxy search; CEU genotype; 1000 Genomes Pilot 1 data set) [75]. We mapped all identified SNPs to UCSC known genes (NCBI36/hg18 assembly, Mar. 2006) using ANNOVAR [52]. Predefined regulatory regions for Yale TFBS, histone modifications defined by ENCODE project, and 11 regulatory elements were extracted from the UCSC database (Additional file 3). Transcription factors (*AR, ER and FoxA1*) binding sites from previous studies [50,51] were also applied for functional annotation [76].

### Functional enrichment of the PCa candidate gene set

The PCa associated gene list was obtained according to annotation results as followed, if the SNP was located in the gene region, then the corresponding gene was selected. Otherwise, we mapped the SNPs to collected eQTL data, which was built based on a set of previously published papers [18,19,21-26,29,30,34] and a web-based database, SCAN [27], to discover the target genes.

To study the functional roles of the gene list, GeneGO database was used for Gene Ontology (GO) and pathway enrichment analysis. The significance of the enrichment (*p*-value) was determined by hypergeometric distribution for the probability of finding a set of genes within a given GO term or pathway, in which lower *p*-value indicated higher potential of non-randomness of the finding. The *p*-value was then adjusted by false discover rate (FDR) with a value of 0.05.

### Construction of PCa-specific transcription regulatory networks

To construct the PCa-specific transcription regulatory networks, the algorithms implemented in GeneGO were applied to the PCa associated genes. In our study, transcription regulation and transcription factor networks were constructed, and the generated networks were ranked by statistical significance of enrichment (the *p*-values). For transcription regulation network construction, the transcription factors were added into the initial gene list to build a separate network around each transcription factor. Additional nodes from GeneGO database were extracted in order to make the target network interconnected. Transcription factor network with shortest paths between the transcription factor and the direct receptor was built using the PCa associated genes as seed nodes.

## Competing interests

The authors declare that they have no competing interests.

## Supplementary Material

Additional file 1 **Summary of the linkage disequilibrium (LD) relationship among the identified 1828 PCa SNPs**. Click here for file

Additional file 2 **Functional annotation of the identified 1828 PCa SNPs using UCSC Known Gene of ANNOVAR**. Click here for file

Additional file 3 **Annotation databases used for the bioinformatics analysis of PCa SNPs**. Click here for file

Additional file 4 **Annotation information over the identified 1828 PCa SNPs based on genomic databases, of which 284 SNPs were successfully interpreted**. Click here for file

Additional file 5 **Details of the 10 most significant transcription regulation and transcription factor networks**. Click here for file

Additional file 6 **Statistical information of the network of AML1/ETO fusion protein, IGF-1 receptor, Insulin receptor and IGF-2 receptor. Note, multiple sheets are offered**. Click here for file

Additional file 7 **Summary of the identified 40 SNP blocks of 1154 SNPs which were functionally interpreted with a gene**. Click here for file

## Declarations

The publication costs for this article were funded by the above grants.

This article has been published as part of *BMC Genomics *Volume 14 Supplement 8, 2013: Selected articles from the International Conference on Intelligent Biology and Medicine (ICIBM 2013): Genomics. The full contents of the supplement are available online at http://www.biomedcentral.com/bmcgenomics/supplements/14/S8.
